# Cell-Based Screening Identifies the Active Ingredients from Traditional Chinese Medicine Formula Shixiao San as the Inhibitors of Atherosclerotic Endothelial Dysfunction

**DOI:** 10.1371/journal.pone.0116601

**Published:** 2015-02-20

**Authors:** Xiaofan Wang, Ruowen Zhang, Liqiang Gu, Yuanyuan Zhang, Xu Zhao, Kaishun Bi, Xiaohui Chen

**Affiliations:** 1 School of Pharmacy, Shenyang Pharmaceutical University, Shenyang, China; 2 Department of Pathology, School of Medicine and Health Sciences, University of North Dakota, Grand Forks, United States of America; 3 School of Traditional Chinese Material Medica, Shenyang Pharmaceutical University, Shenyang, China; College of Tropical Agriculture and Human Resources, University of Hawaii, UNITED STATES

## Abstract

In this study, we performed a phenotypic screening in human endothelial cells exposed to oxidized low density lipoprotein (an *in vitro* model of atherosclerotic endothelial dysfunction) to identify the effective compounds in Shixiao San. After investigating the suitability and reliability of the cell-based screening method using atorvastatin as the positive control drug, this method was applied in screening Shixiao San and its extracts. The treatment of n-butanol fraction on endothelial cells exhibited stronger healing effects against oxidized low density lipoprotein-induced insult when compared with other fractions. Cell viability, the level of nitric oxide, endothelial nitric oxide synthase and endothelin-1 were measured, respectively. The assays revealed n-butanol fraction significantly elevated the survival ratio of impaired cells in culture. In parallel, n-butanol fraction exhibited the highest inhibition of inflammation. The generation of prostaglandin-2 and adhesion molecule (soluble intercellular adhesion molecule-1) was obviously declined. Furthermore, n-butanol fraction suppressed the production of reactive oxygen species and malondialdehyde, and restored the activity of superoxide dismutase. Compounds identification of the n-butanol fraction was carried out by ultra high liquid chromatography coupled to quadrupole time-of-flight tandem mass spectrometry. The active ingredients including quercetin-3-O-(2^G^-α-l-rhamnosyl)-rutinoside, quercetin-3-O-neohesperidoside, isorhamnetin-3-O-neohesperidoside and isorhamnetin-3-O-rutinoside revealed the ability of anti-atherosclerosis after exposing on endothelial cells. The current work illustrated the pharmacology effect of Shixiao San and clearly indicated the major active components in Shixiao San. More importantly, the proposed cell-based screening method might be particularly suitable for fast evaluating the anti-atherosclerosis efficacy of Traditional Chinese Medicines and screening out the interesting ingredients of Traditional Chinese Medicines.

## Introduction

Atherosclerosis (AS) is a complicated vascular disorder involving lipid accumulation, cell death, oxidative damage and inflammatory responses in the arterial wall, resulting in heart disease and stroke. Endothelial dysfunction is considered to be an initial step in the pathogenesis of AS [1]. High plasma level of low-density lipoprotein (LDL) could be oxidatively modified to be oxidized LDL (Ox-LDL), which is closely correlated with accelerated AS [2].

When healthy endothelial cell function is impaired by atherogenic risk factor (Ox-LDL) [3], the delicate balance between proliferation and apoptosis would be disordered firstly, which is widely evaluated by cell viability assay (Roche, Sweden) [4]. The imbalance between vasoconstriction and vasodilatation is also triggered by Ox-LDL. It is well-known that nitric oxide (NO) is one of the vasodilators, and endothelin-1 (ET-1) is the main vasoconstrictor, both of which oppositely regulate the enothelial-dependent vasomotion. Thus, the health condition of vasomotion attributed to the regular release of NO and ET-1 [5, 6].

Besides, growing evidence shows a relationship between oxidative stress and endothelial function, and oxidative stress has been recognized as a key mechanism in the development of vascular damage, particularly AS [7]. There are several possible mechanisms for causing the oxidative stress of endothelial function in AS, including enhanced production of reactive oxygen species (ROS) and decreased release of NO, as well as an attenuated antioxidant system (Superoxide dismutase, SOD) [8]. Moreover, oxidation of membrane lipids, one of the primary events in oxidative cellular damage, can be assessed by measurement of malondialdehyde (MDA), a breakdown product of lipid peroxides [9].

Furthermore, endothelial dysfunction accompanied by inflammatory process leads to increased adhesion molecule to the activated endothelium [10]. For example, soluble intercellular adhesion molecule-1 (sICAM-1), acting as a leukocyte adhesion molecule, directly contributes to inflammatory responses within the blood vessel wall by increasing endothelial cell activation and augmenting atherosclerotic plaque formation [11]. Prostaglandin E2 (PGE_2_) is an important mediator of active inflammation along with activation and recruitment of macrophages and mast cells [12]. Therefore, it is crucial to supervise these parameters associated with endothelial cell function at the early stage of AS discovery process.

Shixiao San, originally recorded in *the Complete Collection of Prescriptions* (Taiping Huimin Heji Ju Fang), is getting increasing attention worldwide for explicating pharmacological mechanism [13, 14]. As a well-known TCM formula, Shixiao San has been widely used for the prevention and treatment of cardiovascular disease in modern clinical therapy. Our previous studies have demonstrated that Shixiao San effectively lower the degree of LDL particles [14, 15]. Indeed, high level of plasma LDL results in endothelium-dependent dysfunction, so it is probably that Shixiao San could restore endothelial function, in part, by lowering serum LDL levels. Nevertheless, there are relatively few reports regarding the reliable pharmacological activity of Shixiao San on treating endothelial dysfunction. Besides, complex chemical compositions of Shixiao San make it extremely difficult to evaluate and screen the bioactive ingredients. Hence, a simple and rapid method is urgently needed to illuminate the efficacy and discover the bioactive compounds of Shixiao San against endothelial dysfunction.

A variety of approaches for screening and analysis of the bioactive fractions or compounds in TCM have been developed and improved during the past decades [16]. Researchers tend to isolate chemical compounds from Traditional Chinese Medicines (TCMs) and test them individually on animal models in conventional ways [17]. However, these approaches are time consuming, arduous, and unsuitable for the rapid screening of bioactive compounds of TCMs. Cell-based screening typically refers to a process in which abundant drug candidates are efficiently tested to identify their biological activity through cell assay in additional biological or pharmacological experiments. Compared with the whole animal models, the cellular models based on different diseases and mechanisms are more adaptive to large-scale candidates screening in TCMs, since they have overcome the shortcomings of animal models, such as time consuming, technical complexity, poor repeatability, expensive cost, low throughput and species difference [18, 19]. Therefore, this unbiased screening is expanding rapidly in drug discovery recently [20]. Human endothelial cell line EA.hy926 is commonly accepted as a tool in exploring the pathogenesis of cardiovascular diseases [21]. To accomplish this study, Ox-LDL exposing on EA.hy926 was carried out as an *in vitro* pathological model for high throughput screening. Through evaluating the parameters above, interesting fraction and compounds from Shixiao San that regulate endothelial cell functions would be rapidly tested *in vitro* in preclinical models.

Herein, we constructed a novel and rapid cell-based screening strategy to systematically explore compounds of Shixiao San regarding their efficacy against atherosclerotic endothelial dysfunction. Compounds identification was carried out by ultra high liquid chromatography coupled to quadrupole time-of-flight tandem mass spectrometry (UHPLC/Q-TOF MS). This method not only offered evidence-based data about the therapeutic mechanism of Shixiao San in molecular level, but also screened out the anti-atherosclerotic candidate compounds. More importantly, for the first time, a standard operating procedure concerning a cell-based screening method that can rapidly detect the target component from a complex sample of anti-atherosclerotic candidate drugs has been recommended.

## Experimental

### Chemicals, reagents and materials

Ox-LDL, human (Yiyuan Biotechnologies, China) was stored at 4°C, and the stock solution was freshly prepared in phosphate-buffered saline before applied to the cultures with a final concentration of 100 μg/mL. Dulbecco’s modified eagle’s medium (DMEM) and fetal bovine serum (FBS) were purchased from Hyclone (Logan, UT, USA). 3-(4, 5-dimethylthiazol-2-yl)-2, 5-diphenyltetrazolium bromide (MTT), dimethyl sulphoxide (DMSO), penicillin and streptomycin were obtained from Sigma (St. Louis, MO, USA). The commercial kits used in biochemical assays of MDA, SOD and NO were purchased from Nanjing Jiancheng Bioengineering Institute, (Nanjing, China). Human enzyme-linked immusorbent assay (ELISA) Kits for measurement of endothelial nitric oxide synthase (eNOS), ET-1, PGE_2_ and sICAM-1 contents were from Senxiong Biological Limited Corporation (Shanghai, China). The raw materials of *Typhae Pollen* and *Faeces Trogopterori* were purchased from Tong-Ren-Tang TCM store (Shenyang, China). Atorvastatin (Atv) was provided by Pfizer. The reference standard of isorhamnetin-3-O-(2^G^-α-L-rhamnosyl)-rutinoside was obtained from National Institute for Food and Drug Control (Beijing, China). Isorhamnetin-3-O-neohesperidoside was purchased from Aladdin Reagent Inc. Isorhamnetin-3-O-rutinoside was from Chengdu Pufei De Biotech Co., Ltd. Quercetin-3-O-(2^G^-α-l-rhamnosyl)-rutinoside, quercetin-3-O-neohesperidoside, kaempferol-3-O-(2^G^-α-L-rhamnosyl)-rutinoside and kaempferol-3-O-neohesperidoside were isolated in our laboratory. Distilled water prepared with demineralized water was employed throughout the experiment. Acetonitrile of HPLC grade was from Fisher Scientific (Fair Lawn, NJ, USA). Formic acid of HPLC grade was provided by Shandong Yuwang Industrial Co., Ltd. (Yucheng, China).

### Preparation of Shixiao San fractions

The method for preparing Shixiao San has been described previously [14]. Then, The crude extract of raw medicinal materials was suspended in water and successively partitioned with petroleum ether (PE), methylene chloride (CH_2_Cl_2_), ethyl acetate (EtOAc) and n-butanol (BuOH) to afford PE, CH_2_Cl_2_, EtOAc and BuOH fractions as well as an H_2_O residue, followed by freeze-drying procedure. The freeze-dried powders were dissolved with DMEM separately and filtrated with 0.22μm cellulose acetate membrane.

### Experimental design

EA.hy926 cells were obtained from the American Type Culture Collection (Manassas, VA, USA). The cells were maintained in DMEM containing 1 g/L glucose supplemented with 10% FBS, 100 U/mL penicillin and 100 μg/mL streptomycin at 37°C in 10% CO_2_. The cells were seeded into plates and randomly assigned into nine groups as described below containing six parallel samples per group. In group III-VII;, final concentrations of Shixiao San and other fractions in all the assays were equivalent to 100 mg/L crude extract of raw medicinal materials. In group IX, final concentration of Atv in the assay was 5 mg/L.

Control group Cells were first incubated with blank medium for 12 h, then incubated with the replaced blank medium for another 12 h.

Model group Cells were first incubated with the medium containing Ox-LDL for 12 h, then incubated with the replaced medium containing Ox-LDL for another 12 h.

Shixiao San group Cells were first incubated with the medium containing Ox-LDL for 12 h, then incubated with the replaced medium containing Ox-LDL and Shixiao San for another 12 h.

PE group Cells were first incubated with the medium containing Ox-LDL for 12 h, then incubated with the replaced medium containing Ox-LDL and PE fraction for another 12 h.

CH_2_Cl_2_ group Cells were first incubated with the medium containing Ox-LDL for 12 h, then incubated with the replaced medium containing Ox-LDL and CH_2_Cl_2_ fraction for another 12 h.

EtOAc group Cells were first incubated with the medium containing Ox-LDL for 12 h, then incubated with the replaced medium containing Ox-LDL and EtOAc fraction for another 12 h.

BuOH group Cells were first incubated with the medium containing Ox-LDL for 12 h, then incubated with the replaced medium containing Ox-LDL and BuOH fraction for another 12 h.

H_2_O group Cells were first incubated with the medium containing Ox-LDL for 12 h, then incubated with the replaced medium containing Ox-LDL and H_2_O fraction for another 12 h.

Atv group (positive control) Cells were first incubated with the medium containing Ox-LDL for 12 h, then incubated with the replaced medium containing Ox-LDL and Atv fraction for another 12 h.

After treatment, the cells were assigned to analysis of cell viability assay, level of intracellular ROS, MDA and SOD assays and extracellular NO, eNOS, ET-1, PGE_2_ and sICAM-1 assays. After investigating the suitability and reliability of the method using Atv as positive control, each group was examined using the assays for its anti-atherosclerotic activities, then the fraction with high activity was selected for further identification using an UHPLC/Q-TOF MS system. In order to test whether the identified compounds have biological activity, the compounds which have reference standards in candidate fraction were given individually test with in the Ox-LDL mediated assay. Therefore, any interesting compounds from Shixiao San that regulate endothelial cell functions would be possible to test in this method.

### Cell viability assay and intracellular ROS measurement

After drug treatment as described in section 2.3, the stock MTT solution was added to all wells of the assay, and plates were incubated 4 h. Afterwards the supernatant was discarded and 150 μL of DMSO per well was added to solubilize formazan crystals for 10 min on a shaker. The optical density was measured by a microplate reader (3001, Thermo Scientific, Finland) at a wavelength of 490 nm.

The measurement of intracellular ROS was based on ROS-mediated conversion of non-fluorescent 2, 7-dichlorofuorescin diacetate (DCFH-DA) into DCFH [22]. The cells were washed with PBS and then incubated with DCFH-DA (10 μM) and DNA stain Hoechst 33342 (10 mmol/L) at 37°C for 30 min. At the end of incubation, the DCFH fluorescence of the cells from each well was measured at an emission wavelength of 530 nm and an excitation wavelength of 485 nm with a FLX 800 microplate fluorescence reader (Biotech Instruments Inc., USA). The background was obtained from cell-free conditions and the results were expressed as the percentage of control (non-stimulated cells) fluorescence intensity. The representative pictures were evaluated by TCS NT Sp5 LSCM instrument (Leica, Germany).

### Assessment of intracellular MDA and SOD

The cells were washed with ice-cold PBS and centrifuged at 1000 r at 4°C for 10 min. The pellets were resuspended with 1000 μL of PBS, freeze-thawed twice at −20°C and centrifuged at 10,000r at 4°C for 15min. The supernatant was collected for MDA and SOD assays, according to the instructions of Nanjing Jiancheng Bioengineering Institute (Nanjing, China), and the activities of enzymes were expressed as units per milligram protein. Protein concentration was determined by the bicinchoninic acid (BCA) method, using BSA as a reference standard.

### Measurement of extracellular NO, eNOS, ET-1, PGE_2_ and sICAM-1

The cultured medium was collected and centrifuged for 15 min at 4000 r 4°C, and then the supernatants were used for the following analyses. The total amount of NO was assessed by using a colorimetric assay kit. Human ELISA kits from Senxiong Biological Limited Corporation (Shanghai, China) were employed for the measurement of eNOS, ET-1, PGE_2_ and sICAM-1 separately in cultured medium.

### Determination of fraction by UHPLC/Q-TOF MS

Each fraction was examined using the assays for its anti-atherosclerotic activities and then the fraction with high activity was selected for further characterization using UHPLC/Q-TOF MS system. UHPLC/Q-TOF MS analytical procedures were performed on an Accurate-Mass Q/TOF 6520 mass spectrometer with an Agilent 1290 LC system (Agilent, USA). The software Mass Hunter workstation was applied to system operation and data collection. The LC separation was achieved on a Zorbax Eclipse Plus C_18_ column (100 mm × 2.1mm, 1.8 μm). The mobile phase consisted of A (0.01% formic acid in water) and B (0.01% formic acid in methanol). The following gradient program was used: 0–7min, 28% B; 7–20 min, 28–45% B; 20–40 min, 45–80% B. The solvent flow rate was 0.2 mL/min. For all UHPLC/Q-TOF MS experiments, the Q-TOF mass spectrometer was operated in the negative ion mode with an electropray ionization source (ESI^-^). The scan range was set at m/z 100–900. Instrument calibration was performed with a sodium formate solution consisting of 10 mM sodium hydroxide in isopropanol/0.2% formic acid (1:1, *v/v*).

Constituents were identified by comparing their retention time, mass value and MS/MS fragmentation with the corresponding parameters of the reference standards. The constituents, lack of reference standards, were identified by comparing the accurately measured mass value and MS/MS fragments with the value reported in references. Finally, the compounds which have reference standards in candidate fraction were given individually in the Ox-LDL mediated assay.

### Statistical analysis

All data was expressed as mean ± standard deviations (SD) and statistical analysis was performed using SPSS software package (version 16.0, SPSS Inc., Chicago, IL, USA). The data following a normal distribution was evaluated by Shapiro-Wilk test. The differences were analyzed by one-way analysis of variance (ANOVA), followed by post hoc analysis with Tukey-Kramer test [23]. Statistical significance was considered when the value of *p < 0.05, and **p < 0.01 indicated highly significant.

## Results and Discussion

### Concentration-dependent viability losses in EA.hy926 cells induced by Ox-LDL

We first carried out the concentration-dependent study of viability losses in EA.hy926 cells induced by Ox-LDL. Medium containing different concentrations of Ox-LDL (0, 25, 50, 100, 200, 300 and 400 μg/mL) was added to find the most appropriate concentration in the model group. The cells were incubated for 24 h followed by observation under an inverted microscope and measurement by MTT assay. The variation of cell viability and the morphological evaluation displayed an Ox-LDL dose-dependent viability loss in EA.hy926 cells. At the concentration of 0, 25, 50, 100, 200, 300 and 400 μg/mL, the cell viability was 100 ± 0.00%, 85.36 ± 3.63%, 79.61 ± 2.18%, 53.89 ± 3.81%, 35.20 ± 1.94%, 15.54 ± 0.54% and 13.78 ± 0.31%, respectively. In summary, 100 μg/mL of Ox-LDL was determined to be the most appropriate concentration which would be applied in the model groups.

### The cell viability change after treatment of Shixiao San and its extracts

**Fig 1:**
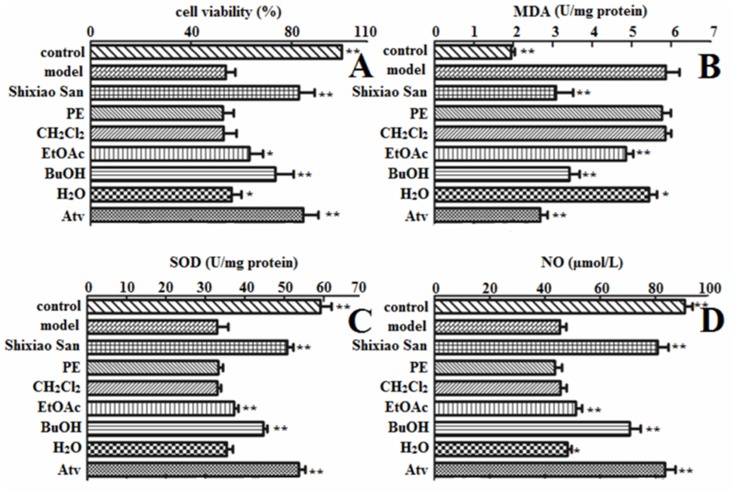
Effect of Shixiao San and its exracts on OX-LDL-induced endothelial cell viability (A) and the level of MDA (B), SOD (C) and NO (D). EA.hy926 cells were exposed to Ox-LDL of 100 μg/mL, and treated with different samples for another 12 hours. All of the data are expressed as the means ± S.D. (*n* = 6). *P < 0.05, ** *p* < 0.01, compared with the model group.

The cell viability carried out by MTT was significantly decreased after Ox-LDL exposure. As shown in Fig. 1A, the significant increased cell viability (compared with model group) indicated that Shixiao San effectively protected cells from Ox-LDL induced damage. The result was similar to that of Atv group. Following further treatment of different extracts, the BuOH group showed a significant improvement over the other fraction groups in cell growth. The cell viability reached up to 73.57 ± 7.34% when cells treated with BuOH fraction, while the cell viability of cells treated with PE, CH_2_Cl_2,_ EtOAc and H_2_O fraction were 52.70 ± 4.32%, 52.98 ± 5.16%, 63.21 ± 5.46% and 56.22 ± 3.88%, respectively.

### Intracellular ROS of Shixiao San and its extracts

**Fig 2:**
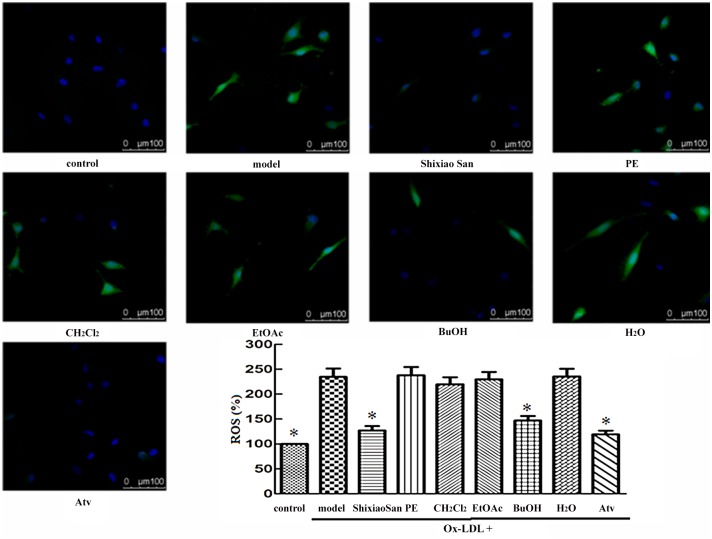
Inhibitory effects of Shixiao San and its exracts on the production of intracellular ROS. EA.hy926 cells were exposed to Ox-LDL of 100 μg/mL, and then treated with different samples for another 12 hours. All of the data are expressed as the means ± S.D (*n* = 6). * *P* < 0.01, compared with the model group.

The up-regulation of ROS in vascular lesions will exert detrimental effects including peroxidation of membranes lipids, endothelium-derived enzymes inactivation and apoptotic occurrences, etc. We can evaluate the intracellular ROS concentration through observing the intensity of fluorescence [8]. Fig. 2 shows the representative picture of the fluorescence in EAhy.926 cells during the various treatments. When incubated in the medium for 30 min, a sudden increment in fluorescence intensity of cells with Ox-LDL indicated the increasing of intracellular radicals. Whereas the increased fluorescence was significantly reduced when cells incubated with Shixiao San and Atv. The effect of BuOH group was similar to that of Shixiao San. However, there is no significant difference in other groups compared with model groups.

### Effect of Shixiao San and its extracts on SOD activity and MDA level

MDA is frequently used as an indicator of tissue damage. The radical formation evaluated as MDA, is shown in Fig. 1B. Shixiao San and Atv significantly attenuated Ox-LDL induced changes of MDA. At the same time, the increased level of MDA induced by Ox-LDL was inhibited by EtOAc and BuOH group. Specifically, the protective effect of BuOH group was better than those of other extracts. As shown in Fig. 1C, the incubation of the cells with Ox-LDL for 24 h caused a significant decrease in SOD activity compared with control group. In contrast, the incubation with Shixiao San, BuOH extract, EtOAc extract and Atv significantly decreased SOD activity. The results suggested that the mechanism of the therapeutical effect of Shixiao San is partly due to antioxidant activity.

### eNOS expression and NO, ET-1 release from endothelial cells

**Fig 3:**
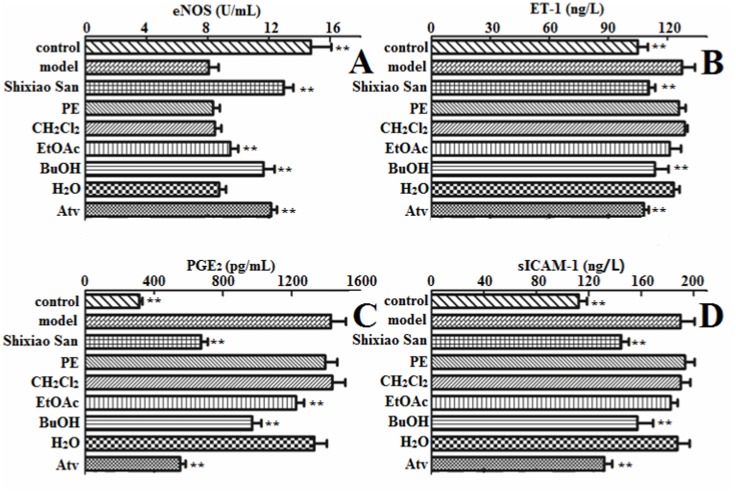
The level of eNOS (A), ET-1 (B), PGE2 (C) and sICAM-1 (D) in the medium with ELISA. EA.hy926 cells were exposed to Ox-LDL of 100 μg/mL, and then treated with different samples for another 12 hours. All of the data are expressed as the means ± S.D (*n* = 6). *P < 0.05, ** *p* < 0.01, compared with the model group.

The eNOS is physiologically pivotal for vascular homeostasis, keeping the vasculature dilated, protecting the intima from platelet aggregates and leukocyte adhesion, and preventing smooth muscle proliferation. eNOS-derived NO is produced by the vascular endothelium under basal conditions. Its production is stimulated by a variety of receptor agonists as well as the shear stress produced by the flowing blood. NO released by endothelial cells is a major endogenous vasodilator system counterbalancing the vasoconstriction produced by the sympathetic nervous system and the rennin-angiotensin system. Thus, eNOS and NO play significant roles in the development of AS [24]. Exposure of EA.hy926 cells to Ox-LDL for 24 hours significantly lessened NO release from endothelial cells (Fig. 1D). Accordingly, NO production of Shixiao San, BuOH, EtOAc, H_2_O and Atv groups were significantly augmented (up to 178%) when compared with that of model group. In contrast, PE and CH_2_Cl_2_ groups had no significant effect on NO production. Furthermore, as depicts in Fig. 3A, Shixiao San led to a significantly enhanced eNOS activity. The activity increased up to 161% compared with model group. From the extracts, BuOH and EtOAc groups increased eNOS promotor activity significantly.

Fig. 3B demonstrates that the exposure to Ox-LDL led to an elevation of ET-1, and the Shixiao San group inhibited the secretion of ET-1 to 87% of the model group. Furthermore, BuOH group showed the ability to reduce the secretion of ET-1 in endothelial cells. In contrast, this decline of ET-1 production was not observed for other groups. Herein, Shixiao San and BuOH extract might regulate imbalances between NO and ET-1 to keep vascular homeostasis.

### Detection of PGE_2_ and sICAM-1 release in cell culture medium

PGE2 is widely recognized as a mediator of inflammation, capable of recruiting proinflammatory cells and causing pain [25]. Fig. 3C showed that Ox-LDL can up-regulated PGE2 expression. On the other hand, the expression of PGE2 was decreased by Shixiao San and Atv groups. Interestingly, the BuOH fraction exhibited a greater inhibition of PGE_2_ to that of the model group.

To determine the effect of Shixiao San and its extracts on the expression of adhesion molecules, we monitored the expression of sICAM-1. Presence of endothelial dysfunction was confirmed by the up-regulation of sICAM-1 in model group compared to the control (Fig. 3D). Meanwhile, level of sICAM-1 showed decreased tendency in Shixiao San and BuOH groups (**p ≤ 0.01). The results exhibited that Shixiao San could weaken inflammatory reaction effectively.

### Identification of anti-atherosclerotic candidate ingredients by UHPLC/Q-TOF MS

**Fig 4:**
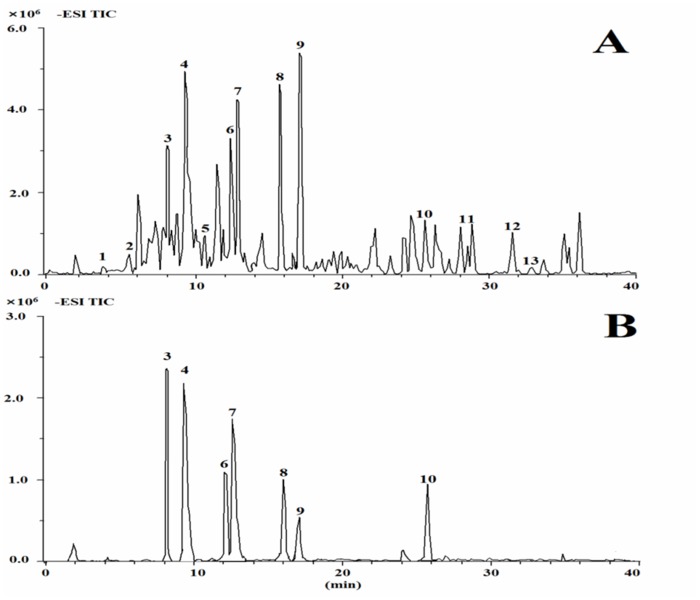
The representative total ion chromatograms of the BuOH fraction (A) and the reference standards (B) in negative mode. (1) 3,3’-methyl quercetin-4’-glucoside, (2) kaempferol-3-O-glucoside/ kaempferol-3-O-galactoside, (3) quercetin-3-O-(2^G^-α-l-rhamnosyl)-rutinoside, (4) quercetin-3-O-neohesperidoside, (5) kaempferol-3-O-glucoside/ kaempferol-3-O-galactoside, (6) kaempferol-3-O-(2^G^-α-l-rhamnosyl)-rutinoside, (7) isorhamnetin-3-O-(2^G^-α-l-rhamnosyl)- rutinoside, (8) kaempferol-3-O-neohesperidoside, (9) isorhamnetin-3-O-neohesperidoside, (10) isorhamnetin-3-O-rutinoside, (11) 5,8-dimethoxy-7-hydroxyflavanone, (12) quercetin-3-O-glucoside, (13) quercetin-3,3’-dimethylether.

**Fig 5:**
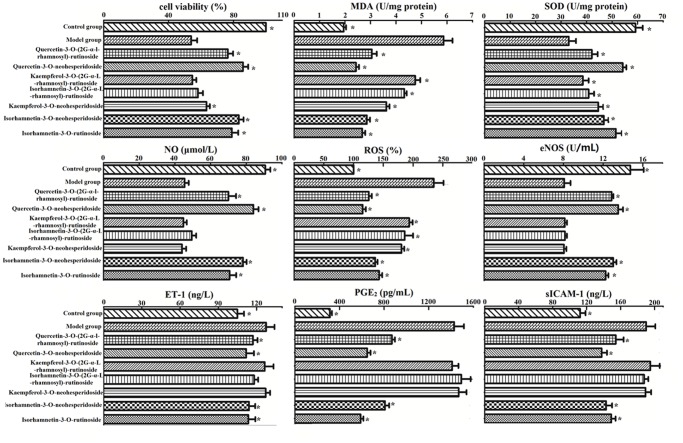
Effect of anti-atherosclerotic candidate ingredients on OX-LDL-induced endothelial cell viability and the level of ROS, MDA, SOD, NO, eNOS, ET-1, PGE2 and sICAM-1. EA.hy926 cells were exposed to Ox-LDL of 100 μg/mL, and treated with different samples for another 12 hours. All of the data are expressed as the means ± S.D. (n = 6). *P < 0.01 compared with the model group.

Collectively, these results demonstrate that Shixiao San possess therapeutic action against Ox-LDL insult. BuOH fraction outperformed other fractions in most of the tests, suggesting that the constituents of BuOH fraction might contribute to Shixiao San’s activities. BuOH fraction was subjected to UHPLC/Q-TOF MS, and thirteen distinct peaks were identified. The representative chromatograms of BuOH fraction were demonstrated in Fig. 4. The information about the analyzed and identified compounds is summarized in Table 1. Among the thirteen identified compounds, there were seven compounds (No. 3, 4, 6, 7, 8, 9, 10) had reference standards and they were more abundant than others that have been identified. Our previous experiments demonstrated that the concentration of the seven compounds presented in crude extract were 2.51, 4.69, 2.97, 3.53, 4.20, 5.54 and 0.58 mg/100mg for quercetin-3-O-(2^G^-α-l-rhamnosyl)–rutinoside, quercetin-3-O-neohesperidoside, kaempferol-3-O-(2^G^-α-L-rhamnosyl)–rutinoside, isorhamnetin-3-O-(2^G^-α-L-rhamnosyl)-rutinoside, kaempferol-3-O-neohesperidoside, isorhamnetin-3-O-neohesperidoside and isorhamnetin-3-O-rutinoside, respectively. The total of them is about 36.85 μmol/L in the assay. In addition, the literature reflected that the concentrations of flavonoids in cell assays were 10–50 μmol/L [26]. Therefore, we selected the concentrations of 5–60 μmol/L to carry out the concentration-dependent study of viability losses in EA.hy926 cells. Every compound reached the maximum of cell viability at 40 μmol/L. Therefore, the seven compounds in candidate BuOH fraction were given individually at the concentration of 40 μmol/L in the Ox-LDL mediated assay. Our studies have shown that four compounds, including quercetin-3-O-(2^G^-α-l-rhamnosyl)-rutinoside, quercetin-3-O-neohesperidoside, isorhamnetin-3-O-neohesperidoside and isorhamnetin-3-O-rutinoside, were particularly potent in inhibiting the concentration of intracellular ROS, suppressing the production of MDA, restoring the activities of SOD, and strongly increasing the level of NO and eNOS (Fig. 5). In parallel, the four compounds exhibited the highest inhibition of inflammation. The generation of PGE_2_ and sICAM-1 were obviously declined.

**Table 1:** List of the retention time and MS data (m/z) for each analyte identified in the BuOH fraction.

No	t_R_ (min)	Assigned identity	ESI^-^ Measured mass/main fragment ions (m/z)	ESI^-^ Calculated mass	ppm
1	3.72	3,3’-methyl quercetin-4’-glucoside	491.1196 [M-H]^-^, 476.1139 [M-H-CH_3_]^-^, 339.1180 [M-H-C_7_H_4_O_4_]^-^, 329.1102 [M-H-glc]^-^, 284.1123 [M-H-C_10_H_7_O_5_]^-^	491.1195	0.2
2	5.58	kaempferol-3-O-glucoside/kaempferol-3-O-galactoside	447.0945 [M-H]^-^, 284.0933 [M-H-glc]^-^, 354.1011 [M-H-C_6_H_5_O]^-^, 295.0930 [M-H-C_7_H_4_O_4_]^-^	447.0933	2.6
3	8.09	quercetin-3-O-(2^G^-α-l-rhamnosyl)- rutinoside	755.2035 [M-H]^-^, 300.2038 [M-H-rha-glc-rha]^-^, 271.2037[M-2H-rha-glc-rha-CO]^-^, 255.2002 [M-2H-rha-glc-rha-CO_2_]^-^, 151.2101 [M-rha-glc-rha-C_8_O_3_H_6_]^-^	755.2040	0.7
4	9.26	quercetin-3-O-neohesperidoside	609.1454 [M-H]^-^, 300.1432 [M-H-rha-glc]^-^, 271.1465 [M-2H-rha-glc-CO]^-^, 255.1442 [M-2H-rha-glc-CO_2_]^-^, 151.1465 [M-rha-glc- C_8_O_3_H_6_]^-^	609.1461	-1.1
5	10.06	kaempferol-3-O-glucoside/kaempferol-3-O-galactoside	447.0943 [M-H]^-^, 284.1031 [M-H-glc]^-^, 354.1015 [M-H-C_6_H_5_O]^-^, 295.0932 [M-H-C_7_H_4_O_4_]^-^	447.0933	2.2
6	12.28	kaempferol-3-O-(2^G^-α-l-rhamnosyl)-rutinoside	739.2101 [M-H]^-^, 284.2232 [M-H-rha-glc-rha]^-^, 255.2139 [M-2H-rha-glc-rha-CO]^-^, 151.2085 [M-rha-glc-rha–C_8_O_2_H_6_]^-^	739.2091	1.4
7	12.98	isorhamnetin-3-O-(2^G^-α-l-rhamnosyl)- rutinoside	769.2194[M-H]^-^, 314[M-H-rha-glc-rha]^-^, 285[M-2H-rha-glc-rha-CO]^-^, 151 [M—rha-glc-rha-C_9_O_3_H_8_]^-^	769.2197	-0.4
8	15.84	kaempferol-3-O-neohesperidoside	593.1503 [M-H]^-^, 284.1324 [M-H- glc-rha]^-^, 255.1588 [M-2H-glc-rha-CO]^-^, 151.1493 [M-glc-rha-C_8_O_2_H_6_]^-^	593.1512	-1.5
9	17.13	isorhamnetin-3-O-neohesperidoside	623.1619 [M-H]^-^, 314.1687 [M-H- glc-rha]^-^, 285.1546 [M-2H- glc-rha-CO]^-^, 151.1612 [M- glc-rha-C_9_O_3_H_8_]^-^	623.1618	0.2
10	25.83	isorhamnetin-3-O-rutinoside	623.1617 [M-H]^-^, 314.1685 [M-H-glc-rha], 285.1544 [M-2H-glc-rha-CO]^-^, 151.1614 [M-glc-rha-C_9_O_3_H_8_]^-^	623.1618	-0.2
11	28.76	5,8-dimethoxy-7-hydroxyflavanone	299.0939 [M-H]^-^, 299.0723 [M-H_2_O]^-^, 251.0910 [M-CH_5_O_2_]^-^, 195.0893 [M-C_8_H_9_]^-^	299.0925	4.7
12	31.65	quercetin-3-O-glucoside	463.0871 [M-H]^-^, 300.0933 [M-H-gal]^-^,271.0867 [M-_2_H-gal-CO]^-^, 255.0435 [M-_2_H-gal-CO_2_]^-^, 151.0785 [M- gal-C_8_O_3_H_6_]^-^	463.0882	-2.4
13	32.87	quercetin-3,3’-dimethylether	329.0653 [M-H]^-^, 314.0938 [M-H-CH_3_]^-^, 299.1021 [M-H-C_2_H_6_]^-^, 206.0969 [M-H-C_7_H_7_O_2_]^-^, 178.1016 [M-H-C_10_H_10_O_3_]^-^	329.0667	-4.3

### Discussion

To exclude the possibility of cytotoxicity caused by Shixiao San and its extracts treatment, MTT assay was performed in EA.hy926 cells treated with Shixiao San and each extract for 12 h. There’s no significant variation between sample groups and control groups in cell viability, indicating that the therapeutic effect of Shixiao San extracts on endothelial cells treated by Ox-LDL has none business with the potential cytotoxic action. The report indicated that Atv exerted many favorable effects on the endothelium and effectively attenuate endothelial dysfunction in the presence of atherosclerotic risk factors (Ox-LDL). Therefore, we selected Atv as positive control to confirm the suitability and reliability in cell-based screening system, and the results corresponded with the study of S. Wolfrum [27].

We systematically analyzed the correlations between Shxiao San and its different fraction extracts. BuOH extract that mainly contains flavonoids dominantly augmented NO output and attenuated ET-1 to maintain a delicate balance in the vasculature between vasodilation and vasoconstriction. Increased active eNOS levels may antagonize the development of endothelial dysfunction and subsequent AS. Moreover, the viability loss in cells induced by Ox-LDL was markedly restored as measured by MTT assay. Furthermore, the oxidative damage was repaired by some free-radical scavengers, such as SOD, and this effect was accompanied by suppressing the production of MDA and intracellular ROS. Leukocyte adhesion is mediated by inducible cell adhesion molecules such as ICAM-1, expressed at high levels on the surface of activated endothelial cell. Shixiao San and BuOH fraction demonstrated anti-inflammatory effect through attenuating the expression of sICAM-1 and PGE_2_. In summary, the excellent regulate functions confirmed the pharmacological effect of Shixiao San and clearly indicate that BuOH extract could be a potential anti-atherosclerosis fraction.

In the present study, we discovered four novel AS therapeutic flavonoids on Ox-LDL induced endothelial dysfunction, an early event in the development of AS.

**Fig 6:**
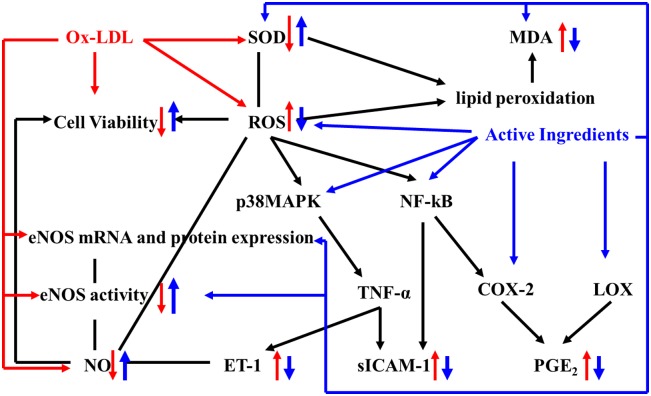
The schematic to disclose the damage mechanism of Ox-LDL (red) and the therapeutic mechanism of Shixiao San and active ingredients (blue) in molecular level.

Fig. 6 is the schematic to reveal the damage mechanism of Ox-LDL and the therapeutic mechanism of active ingredients in molecular level. Oxidative stress, which is induced by ROS, is known to play a critical role in endothelial dysfunction. According to the result of ROS section, we speculated that the underlying mechanism of antioxidant capacity may be associated with their inhibiting or scavenging production of ROS. Moreover, the decrease of MDA proved the recession of lipids peroxidation. Thus, the four flavonoids may protect cells from the damage effect of oxygen radicals or eliminate production of ROS. Furthermore, compounds restored the activity of SOD, which demonstrated that they may enhance cellular antioxidant defense to exert their powerful antioxidant capacity. In addition, modern pharmacological studies confirmed that quercetin-3-O-neohesperidoside, isorhamnetin-3-O-neohespeeridoside and isorhamnetin-3-O-rutinoside have the potent antioxidant activity [28–31], which supported our ideas. *In vitro*, oxidative metabolites are potent inducers of endothelial cell death [32]. Here, we demonstrated that Ox-LDL, a precursor of other ROS, can significantly lead to massive endothelial cells apoptosis as evidenced by cell viability. Consistent with these reports, we hypothesized that the high anti-apoptotic activities of these four flavonoids in the vascular endothelial cells treated by Ox-LDL may be associated with their powerful antioxidant capacity.

Previous study has shown that flavonoids could increase the mRNA and protein expression of eNOS in rats [33]. Therefore, these four flavonoids may increase the expression to promote the growth of eNOS. In addition, the results reflected that these four flavonoids might promote the synthesis and release of NO directly in the endothelial cells by activating eNOS. It was reported that the NO synthesized from endothelium could inhibit apoptosis induced by various apoptotic stimuli [34]. There have been several reports linking excess oxidative stress to the impairment of NO production, resulting in decreased NO bioactivity in patients with the cardiovascular diseases [35]. In our study, pre-incubation with these four flavonoids significantly inhibited NO decrease, which may be due to their antioxidant abilities.

PGE_2_ are small lipid molecules derived from arachidonic acid (AA) and are produced by the action of cyclooxygenases (COX-1 and COX-2) and lipoxygenase (LOX) [36]. Different studies have confirmed that flavonoids like quercetin and kaempferol produced a significant concentration-dependent decrease of COX-2 and LOX level [37]. Wogonin and luteolin could inhibit the expression of COX–2 to recover the production of PGE_2_ [38, 39]. Thus, all these mechanisms could partly explain the anti-inflammatory effect (reduce the concentration of PGE_2_) of active compounds. Chen systematic explored the effect of delphinidin on endothelial cell adhesion induced by Ox-LDL and revealed this effect is mediated via ROS/p38MAPK/NF-kB signaling pathway. ROS has a major effect on p38MAPK activation and the release and nuclear translocation of the NF-kB complex. NF-kB plays an important role in the transcriptional regulation of inflammatory proteins such as COX-2 and sICAM-1 [40]. Tumor necrosis factor-α (TNF-α) is regulated through the MAPK pathway, induce sICAM-1 to be shed off from the cell surface of various primary cells and cell lines [39, 41]. In our study, we also found that active compounds attenuated the expression of sICAM-1 concomitantly with reduction of intracellular ROS levels. These findings suggested that the active compounds may attenuate the up-regulated expression of adhesion molecules via inhibiting ROS/p38MAPK/NF-kB pathway. Pro-inflammatory cytokines including TNF-α is known to stimulate ET-1 production in cultured endothelial cells [42]. ROS serves as a major mediator of intracellular signaling of TNF-α [43]. This occurred through inhibiting ROS-independent p38MAPK that regulated TNF-α. In conclusion, it is speculated that the active compounds could down-regulate ET-1 production via the inhibition of ROS in endothelial cells. Therefore active compounds may inhibit ET-1 production through their antioxidant effect.

In the present study, different flavonoids (aglycone and glycoside) exhibited diverse capacity in endothelial cells. Although we did not test a whole battery of flavonoids, we assumed that there may be a relationship between their structure and anti-atherosclerotic activity. The antioxidant actions of flavonoids in oxidant-induced endothelial apoptosis have been shown to be mediated through their H^+^-donating properties, the location and number of—OH are crucial for the antioxidant activity of flavonoids [44]. The article by Yi demonstrated that significant correlations were observed between the number of—OH moieties in B-ring and the inhibitory effects on endothelial dysfunction. Furthermore, 3′, 4’-ortho-dihydroxyl on B-ring appeared to be the main structural requirements for activity [23]. Our results also reflected the poor effect of kaempferol-3-O-(2^G^-α-L-rhamnosyl)-rutinoside and kaempferol-3-O-neohesperidoside (lack of 3′-ortho-dihydroxyl on B-ring). Therefore, 3′, 4′-ortho-dihydroxyl on B-ring should be correlated closely to the inhibitory effect on endothelial dysfunction. Additionally, structurally resembling quercetin-3-O-(2^G^-α-l-rhamnosyl)-rutinoside and isorhamnetin-3-O-(2^G^-α-l-rhamnosyl)-rutinoside containing 3-O-(2^G^-α-l-rhamnosyl)-rutinoside showed weaker effect on cell viability and other indicators than quercetin-3-O-neohesperidoside and isorhamnetin-3-O-neohesperidoside/ rutinoside, respectively. Kaempferol-3-O-(2^G^-α-l-rhamnosyl)-rutinoside showed no notable effect. Compared with neohesperidoside or rutinoside, the glycoside, 3-O-(2^G^-α-l-rhamnosyl)-rutinoside, significantly attenuated the inhibitory effect of flavonoids on endothelial dysfunction. Our work might provide some evidences for AS prevention and a strategy for the design of novel AS preventive agents.

## Conclusion

Through this novel cell-based screening method, we evaluated the effects and mechanisms of Shixiao San and their extracts obtained using PE, CH_2_Cl_2_, EtOAc, EtOAc, BuOH and H_2_O fractions on Ox-LDL activated human endothelial cells. The results demonstrated that the BuOH extract could restore cell viability, protect vascular integrity, suppress oxidative damage and strengthen anti-inflammatory effect of atherosclerotic endothelial dysfunction. Further studies revealed that quercetin-3-O-(2^G^-α-l-rhamnosyl)-rutinoside, quercetin-3-O-neohesperidoside, isorhamnetin-3-O-neohesperidoside and isorhamnetin-3-O-rutinoside from BuOH extract might contribute to the therapeutic potential of AS diseases. Therefore, there is considerable interested in the identification of more potentially active compounds from TCMs to treat diseases involving AS using the novel analytical method in the future.
